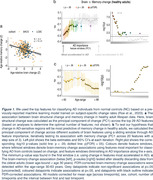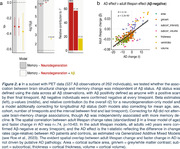# Adult lifespan neurodegeneration is associated with memory decline independent of amyloid deposition

**DOI:** 10.1002/alz.088718

**Published:** 2025-01-09

**Authors:** James M Roe, William J. Jagust, Susan M. Landau, Theresa M. Harrison, Håkon Grydeland, Maksim Slivka, José‐Luis Alatorre‐Warren, Øystein Sørensen, Kristine B Walhovd, Anders M Fjell, Didac Vidal‐Piñeiro, Yunpeng Wang

**Affiliations:** ^1^ LCBC, University of Oslo, Oslo Norway; ^2^ University of California, Berkeley, Berkeley, CA USA; ^3^ Computational Radiology and Artificial Intelligence, Oslo University Hospital, Oslo Norway

## Abstract

**Background:**

Current models of AD posit neurodegeneration and cognitive decline occur downstream in a pathophsyiological cascade initiated by amyloid (Aβ), yet lifespan research suggests the brain regions and cognitive functions impacted most by AD exhibit the steepest, steady decline rates across life. We hypothesised adult lifespan neurodegeneration in AD‐vulnerable brain regions would predict memory decline rates detectable in healthy adults as they age, independent of Aβ.

**Method:**

We combined MRI scans across three large longitudinal cohorts of cognitively healthy adults (age 30‐96 years) to estimate brain change relative to the change expected given a person’s age (2‐14 timepoints; 4125 scans of 1027 individuals; cohorts: LCBC, the Berkeley Aging Cohort Study [BACS]; ADNI [stable cognitively healthy]). We similarly estimated episodic memory change, as measured by the California Verbal Learning Test (LCBC and BACS; 2356 observations of 601 individuals). Using a set of features we previously determined classifies AD patients from controls (AUC=.952), we ran multivariate models to test associations between brain structure change and memory change. Specifically, we calculated the principal component of change across different subsets of brain features using a sliding window through AD feature importance, iteratively testing its association with memory change. In a subset with PET data (537 Aβ observations of 262 individuals), we tested whether brain‐memory change associations were independent of Aβ. Finally, we tested the spatial correlation between adult lifespan brain change rates in only Aβ‐negative individuals (adults >40 years confirmed Aβ‐negative at every timepoint) and an AD‐control effect size based on change.

**Result:**

Associations between brain structure change and memory change were detectable in healthy adult lifespan data (p=7.0e^‐10^), present in comparatively young adults (in 30–65‐year‐olds), and strongest in regions changing faster in AD. Correcting for Aβ did not attenuate brain‐memory change associations, though Aβ was independently associated with more memory decline (p=2.7^‐3^). The spatial correlation between adult lifespan change rates (in Aβ‐negative) and accelerated change in AD was r=.74, p=10^‐63^.

**Conclusion:**

Our results show neurodegeneration in AD‐sensitive regions exists independently of Aβ, and that such changes are not benign, but track with more memory decline across the healthy adult lifespan.